# Involving Stakeholders as Communication Partners in Research Dissemination Efforts

**DOI:** 10.1007/s11606-021-07127-3

**Published:** 2022-03-29

**Authors:** A. Rani Elwy, Elizabeth M. Maguire, Bo Kim, Gavin S. West

**Affiliations:** 1 Center for Healthcare Organization and Implementation Research, VA Bedford Healthcare System, Bedford, MA USA; 2 Department of Psychiatry and Human Behavior, Warren Alpert Medical School, Brown University, Providence, Rhode Island USA; 3 Center for Healthcare Organization and Implementation Research, VA Boston Healthcare System, Boston, MA USA; 4 Department of Psychiatry, Harvard Medical School, Boston, MA USA; 5 VA Salt Lake City Healthcare System, Salt Lake City, UT USA; 6 Office of the Assistant Under Secretary for Health for Clinical Services, Washington, DC USA

## Abstract

Moving evidence into practice requires the support of stakeholders, who are critical actors in the research process. Yet, research teams need strategies for determining who these stakeholders are, what their roles should be, and how to involve them in research and dissemination activities. In this *Perspective*, we discuss steps for identifying, categorizing, and including stakeholders in the research process, as a precursor to involving them as communication partners in research dissemination efforts. Effectively communicating the results of research is critical for increasing stakeholders’ buy-in for the adoption and sustainment of this evidence. However, this communication is best if it comes from the end-users themselves, the stakeholders, who have a specified involvement in the research process. Combining elements from dissemination, implementation, and management science literature, we identify specific tools and strategies for researchers to (1) understand the roles of various stakeholders potentially impacted by their work, and (2) recognize the specific communication activities these stakeholders could be engaged in, to support the dissemination of research findings. We present a 3-Step Plan for identifying, categorizing, and involving stakeholders in the research process in a way that will lead to their role as communication partners when results are ready to be disseminated widely.

Fewer than 50% of clinical innovations become part of routine clinical practice,^1^ and only about 20% of research funding impacts public health.^2^ Dissemination of research evidence and implementation of healthcare innovations are often complex, involving diverse stakeholders.^3,4^ Stakeholders are defined as any individuals, groups, or organizations that can affect, or can be affected by, another individual, group, or organizations.^5^ For healthcare researchers and implementation scientists, stakeholders may include patients and family members, clinicians, administrators, community-based leaders, and policymakers.

Despite increases in researchers’ knowledge of stakeholders’ roles in moving research evidence into routine practice,^6–8^ it is often unclear *how* to identify and include a range of stakeholders in the research process, as a precursor to collaborating with them for research dissemination. By identifying and including stakeholders in research efforts from the beginning, stakeholders can hold peer-to-peer conversations with researchers to build trust in them.^4^ Stakeholders can in turn understand an innovation’s key aspects and reasons to partake in research efforts, setting the stage for their involvement as communication partners in research dissemination efforts. Without this trust, stakeholders are unlikely to engage in the research effort itself and, thus, will not be viable communication partners when it is time to disseminate research efforts.^4,6–8^

## THE 3-STEP PLAN FOR ENGAGING STAKEHOLDERS IN THE RESEARCH PROCESS

Our team turned to the management science literature to find tools to identify stakeholders, understand their positions, and select specific strategies for working with them.^5,9–12^ Merging management science with implementation science, we identified three critical steps for any research team at the start of a project, when beginning stakeholder engagement: (1) ask questions to identify all relevant stakeholders; (2) map stakeholders into one of four specific stakeholder groups; and (3) select specific strategies for engaging each stakeholder group in the research process.

### Ask Questions to Identify Stakeholders

Stakeholder theory specifies stakeholder groups and recommends methods by which managers and leaders (or investigators and project directors in healthcare research and implementation) can consider these groups’ interests.^10,11^ Stakeholder theory proposes questions that any research team can ask themselves at the beginning of a research project: Who are our current/potential stakeholders? What are their interests/rights in our project? How does each stakeholder affect us? What are the potential, immediate (clinical or organization), and downstream (policy) effects of the research on each stakeholder? What assumptions do we make about each stakeholder? Per dissemination and implementation science literature, we can add two questions: What are stakeholders’ alliances with other organizations? Which stakeholders do we consider to be the most credible sources of information to other stakeholders? We recommend answering these questions by completing a stakeholder table, adapted from health sector policy reform efforts (Table 1).^13^

**Table 1:** Stakeholder Identification Table

Current Stakeholders								
Name	Position and Organization	Interests or Rights in Project (Advantages or Disadvantages)	Potential Effects of Stakeholder on Research Effort and Team	Potential Immediate Effects of Research on Stakeholder	Potential Downstream Effects of Research on Stakeholder	Assumptions Team Makes about Stakeholder	Alliances with other Organizations	Credible Source of Information to Others (Yes/No; Explain)
Potential Stakeholders								
Name	Position and Organization	Interests or Rights in Project (Advantages or Disadvantages)	Potential Effects of Stakeholder on Research Effort and Team	Potential Immediate Effects of Research on Stakeholder	Potential Downstream Effects of Research on Stakeholder	Assumptions Team Makes about Stakeholder	Alliances with other Organizations	Credible Source of Information to Others (Yes/No; Explain)

### Stakeholder Mapping

After answering these questions, research teams should start mapping these stakeholders into groups, to assess each stakeholder’s potential to threaten or to cooperate with the research. The terms “threaten” and “cooperate” stem from Stakeholder Theory.^10,11^ Within healthcare research and implementation science, these terms can represent stakeholder’s potential to “impede,” instead of threatening, a research effort; or “collaborate” with a research team, instead of merely cooperating. Stakeholders can be *supportive*, *mixed blessings*, *non-supportive*, or *marginally supportive*.^12^ *Supportive* stakeholders may be non-profit organization leaders with a mission similar to that of the research team, partnering with researchers to bring more visibility to their own activities. Stakeholders viewed as *mixed blessings* may be clinical or organizational leaders who are interested in the healthcare innovation as it may improve the health of the patients they serve but are concerned that the innovation will disrupt clinical workflows and impede process efficiencies. *Non-supportive* stakeholders may be those protecting the interests of their own members, feeling that the research demands too much of their clinical staff, for example. These non-supportive groups might be union leaders, who are concerned that research activities are expanding their members’ scope of work, or those in the media who misrepresent a research endeavor, dampening the public’s trust in it. Finally, *marginally supportive* stakeholders may be patient or consumer groups, who believe in the research but feel that their own perspectives regarding the innovation are not being addressed by the current research process.

### Identifying Engagement Strategies

After mapping potential stakeholders to one of the four groups, the research team can consider strategies for involving them, based on the levels of threats (or impediments) and cooperation (or collaboration) present for each of these groups. Table 2 presents an overview matrix of stakeholder categories by their potential for cooperation with and threat to the research team and research process.^10–12^ Table 2 also provides the strategy that may overcome these threats or impediments and lead to more stakeholder engagement and, eventually, communication and dissemination efforts.

**Table 2:** Overview Matrix Of Stakeholder Categories by the Potential for Stakeholders’ Cooperation with and Threat to the Research Team/Process ^9–12^

**Potential for threat or impediment**			
**Potential for cooperation or collaboration**		**Low**	**High**
	**Low**	Type: Marginal Strategy: Monitor	Type: Non-supportive Strategy: Defend
	**High**	Type: Supportive Strategy: Involve	Type: Mixed blessing Strategy: Collaborate

### Involvement Strategy for Supportive Stakeholders

For supportive stakeholders, the research team’s role is to involve these stakeholders throughout the research activities, to ensure that their support continues and that stakeholders feel that they are important contributors throughout. The research team should regularly check in with these stakeholders, provide updates on progress, and seek their input.

### Collaboration Strategy for Mixed Blessing Stakeholders

When stakeholders present as mixed blessings—those who have not only a high potential for collaboration but also a high potential for impeding the research—the research team must work towards collaboration. The easier tactic would be to ignore these stakeholders, but we implore research teams to take the opposite approach and try to sway these stakeholders to the research team’s position. These stakeholders should be invited to the decision-making table, to have their perspectives weigh in on the researcher’s assessments.

### Defensive Strategy for Non-supportive Stakeholders

The potential for threat or impediment is high among non-supportive stakeholders. Using a defensive strategy involves a research team not only maintaining their ground but also allowing non-supportive stakeholders to bring forth new ideas for the research, such as how, where, or when it is conducted. If clinical or healthcare organizational stakeholders, for example, are concerned about employees involved in the research process, this discussion can alleviate concerns, or it may help the research team to identify new ways of conducting research that addresses these worries.

### Monitor Strategy for Marginally Supportive Stakeholders

For marginally supportive stakeholders, where potential threats/impediments and cooperation/collaboration are low, the research team should consider continuously monitoring these stakeholders’ perspectives. Although they are unlikely to be as large a threat as non-supportive or mixed blessing stakeholders, the research team is missing out on a potential role for this group as research disseminators. One reason that a marginally supportive group, such as a patient advocacy group, does not initially buy into the research process may be because they feel that their own views are not considered important by the research team. Creating a space for these stakeholders to share their perspectives (e.g., community-based forums, advisory boards) will allow researchers to monitor whether these stakeholders’ positions, and thus, levels of threat and cooperation are changing.

## CASE STUDY: ENGAGING STAKEHOLDERS EARLY IN A RESEARCH PROCESS, LEADING TO THEIR ROLE AS COMMUNICATION PARTNERS

The 3-Step Plan can be used for many different types of research and can be particularly useful for complex interventions or when significant changes are made to well-established processes. In this case example, we used this plan to look at a highly publicized process in our healthcare organization, the communication of large-scale adverse events, as required by the Veterans Health Administration (VHA) Directive 1004.08.^14^ Our work sought to change the established communication processes around these events. Large-scale adverse events are unanticipated incidents that occur during the process of patients receiving healthcare, which either lead to multiple patients’ injury or increase their risk of injury, yet are not recognized by the healthcare system at the time of the incident.^15^ Examples of large-scale adverse events include equipment disinfection lapses (e.g., endoscopes, dental equipment), unsafe injection practices (e.g., reuse of single patient syringes), and events related to provider behavior, such as practicing unsafe medicine.^16^

First, our team *asked questions* of a Stakeholder Advisory Board consisting of physicians, nurses, patient safety officers, a former congressional staff member, other government agency employees, and a health communication specialist, established to guide the research process, to identify this directive’s stakeholders. *Identified stakeholders* included patients, family members, frontline clinicians, medical center leaders, quality and safety specialists, and regional and national leaders. After interviewing 97 of these stakeholders, our team answered questions related to those outlined in Table 1. These interviews elucidated the roles that each stakeholder plays in the disclosure process, their perceived advantages and disadvantages of the disclosure directive, and how the directive impacts their work directly, as well as communication gaps during disclosures.^16^

Second, our team then mapped each stakeholder to a stakeholder group. We identified *supportive stakeholders* (patients, family members, patient safety officers, nurses) who wanted disclosure processes to improve, with whom to communicate as early and clearly as possible; *mixed blessing stakeholders*, leaders worried about backlash from the media and congressional representatives when the disclosure news would become public; and *marginally supportive stakeholders*, public affairs officers who were concerned about proactively communicating with the media and congressional representatives because they had never proactively provided information before an inquiry. We did not identify any non-supportive stakeholders.

Third, our team created specific *engagement strategies* for encouraging best practice communication efforts, to implement VHA Directive 1004.08 as intended. Our team created a Large-Scale Disclosure Toolkit that incorporated a range of *involvement*, *collaboration*, and *monitoring strategies*.^17^ The toolkit was disseminated throughout the organization and was then implemented and evaluated in two real-time large-scale adverse event disclosures. Qualitative feedback from these two disclosures was positive, suggesting some additional changes in communication strategies and training efforts, such as ensuring that the toolkit be used flexibly to adapt to every disclosure situation. The toolkit was updated accordingly and was placed on an internal website where facilities could access the information and ask questions of the research team as needed.^17^

### Moving from Research Engagement to Communication Partners in Dissemination Efforts

As diverse stakeholders began to see the impact of the Large-Scale Disclosure Toolkit on their work, the research team was invited to become involved in national disclosure discussions. VHA leaders discussed the toolkit with colleagues and emphasized to others the patient-centered communication principles described in the toolkit. Our research team witnessed this transformation, from the research team communicating about the toolkit to VHA leaders readily communicating about the toolkit to others, without the involvement of the research team. We strongly believe that engaging these stakeholders from the beginning in the research process, learning from them through interviews, mapping stakeholders to specific groups, and identifying strategies to engage them during the disclosure process and development of the toolkit led to stakeholders communicating about the toolkit and disseminating information about it to others in their network.

Importantly, these steps that we took with stakeholders are applicable to research beyond this case study’s particular context of large-scale adverse event disclosure. Given that stakeholder engagement is not often prioritized as a dissemination strategy,^18,19^ research efforts that would most notably benefit from applying these steps would be those that have stakeholders who (1) must be involved in identifying the problem to be solved and (2) are in positions to impact the implementation of the knowledge generated from the research.^19–21^ Especially for such research efforts, not building stakeholder relationships could prevent stakeholders from developing trust in the researchers and the evidence, decreasing their willingness to support research dissemination.^4,6–8^ Furthermore, with strategies for managing stakeholders being specific to each stakeholder group,^11^ not mapping stakeholders to specific groups would be a missed opportunity to optimally select and use stakeholder-facing strategies.

## CONCLUSION

Drawing on dissemination, implementation, and management science literature, we developed a 3-Step Plan to help teams preemptively identify stakeholders relevant to their research mission at the beginning of a study, classify these stakeholders into groups that describe their potential for cooperation with and threat to the research process, and select strategies for maintaining or increasing stakeholders’ support in research activities. In this way, key stakeholders are primed to share critical information about research evidence with their networks, supporting the research mission and eventual public health impact.
